# Takotsubo Syndrome Mimicking Apical Hypertrophic Cardiomyopathy: A Case Report

**DOI:** 10.1002/ccr3.72796

**Published:** 2026-05-22

**Authors:** Benjamin Vögeli, Recep Ali Hacialioglu, Bilal Cevik, Julian Flury, Alvin Oliver Payus, Nisha Arenja

**Affiliations:** ^1^ Department of Cardiology University Hospital Basel Basel Switzerland; ^2^ Department of Internal Medicine Bürgerspital Solothurn Solothurn Switzerland; ^3^ Department of General Internal Medicine University Hospital of Bern Bern Switzerland; ^4^ Department of Medicine, Faculty of Medicine & Health Sciences Universiti Malaysia Sabah Kota Kinabalu Sabah Malaysia; ^5^ Department of Cardiology Solothurner Spitäler AG Olten Switzerland; ^6^ Faculty of Medicine University of Basel Basel Switzerland

**Keywords:** apical hypertrophic cardiomyopathy, cardiac magnetic resonance, case report, endocardial edema, Takotsubo syndrome

## Abstract

Takotsubo syndrome (TTS) is a reversible form of cardiomyopathy that is triggered by emotional or physical stress, leading to a typical image of apical ballooning. In contrast, apical hypertrophic cardiomyopathy (ApHCM) is a condition characterized by thickening of the left ventricular (LV) apical wall and obliteration of the LV apex. Although both conditions result in opposing imaging features, they can significantly overlap, especially in the early stages of the disease. This report describes a case of a 66‐year‐old woman whose initial presentation was consistent with TTS but later raised suspicion of an ApHCM phenotype.

## Key Clinical Message

Takotsubo syndrome and apical hypertrophic cardiomyopathy may mimic each other on ECG and echocardiography. Endocardial edema can confound diagnosis, emphasizing the crucial role of cardiac MRI in differentiating transient TTS from structural ApHCM to avoid misdiagnosis and ensure appropriate management.

## Graphical abstract

Graphical abstract showing possible clinical findings in Takotsubo syndrome and apical hypertrophic cardiomyopathy.

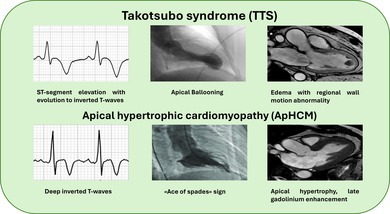

**Abbreviations**
AMI: Acute myocardial infarction
ApHCM: Apical hypertrophic cardiomyopathy
CAD: Coronary artery disease
CMR: Cardiac magnetic resonance imaging
ECV: Extracellular volume
HCM: Hypertrophic cardiomyopathy
LV: Left ventricle
LVEF: Left ventricular ejection fraction
MINOCA: Myocardial infarction with nonobstructive coronary arteries
NSTEMI: Non‐ST‐elevation myocardial infarction
TTE: Transthoracic echocardiography
TTS: Takotsubo syndrome

## 1 Introduction

Takotsubo syndrome (TTS), often precipitated by emotional or physical stress, presents with clinical features resembling those of acute myocardial infarction (AMI), but without evidence of acute coronary thrombosis [1]. A defining feature of TTS is transient apical ballooning of the left ventricle, believed to result from a surge in catecholamines. The condition is typically reversible, with approximately 90% of patients demonstrating full recovery [2].

Apical hypertrophic cardiomyopathy (ApHCM), by contrast, is characterized by myocardial thickening and possible obliteration of the left ventricular apex—findings that distinguish it from the apical ballooning observed in TTS. We report the case of a woman who presented with chest pain and an initial clinical picture suggestive of TTS. However, subsequent evaluation raised the possibility of underlying ApHCM.

## 2 Case History/Examination

A woman, 66 years of age, with a history of hypertension presented to the emergency department with chest pain and general weakness lasting for a few hours. On initial presentation the patient was hemodynamically stable with borderline hypotension of 100/50 mmHg, heart rate of 83/min, and normal oxygen saturation of 97%. During the physical examination, she was in some distress. However, no clinical abnormalities were reported. Besides hypertension, which was treated with valsartan/hydrochlorothiazide 80/12.5 mg once daily, the patient had a positive family history of coronary artery disease (CAD) from the maternal side. The patient reported that she felt unusually weak when she carried bags down a floor. Additionally, she was preparing for a trip and was under significant time pressure. Further questioning revealed an intense psychosocial stress situation due to taking care of her sick mother.

## 3 Differential Diagnosis, Investigations, and Treatment

**FIGURE 1:**
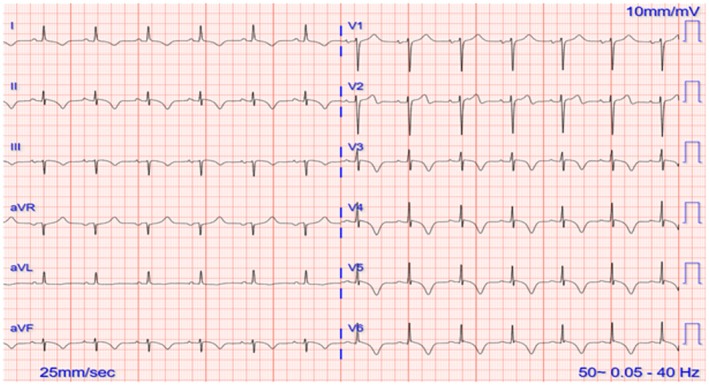
ECG on admission showing marked T wave inversions.

**FIGURE 2:**
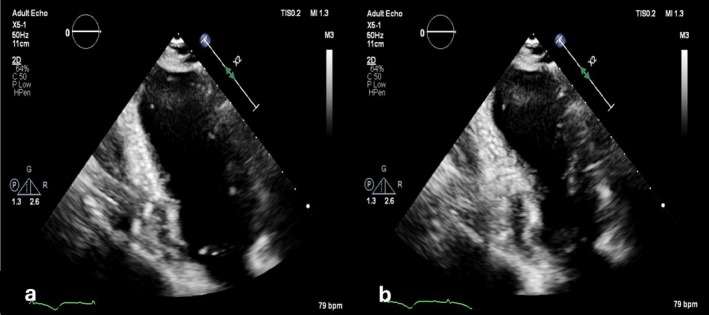
Apical two‐chamber view on 2D echocardiography showing apical akinesis (a: End diastole; b: End systole).

Her initial ECG showed sinus rhythm with left axis deviation and prolonged QTc interval with marked profoundly inverted T waves in the anterior and inferior leads (Figure 1). The 2D transthoracic echocardiography (TTE) showed a moderately reduced LV ejection fraction (LVEF) of 35% due to akinesia of all apical segments (Figure 2). Her high‐sensitive troponin T levels were elevated to 384 ng/L. The rest of the blood tests were normal except for a mildly elevated serum creatinine of 121 μmol/L.

**FIGURE 3:**
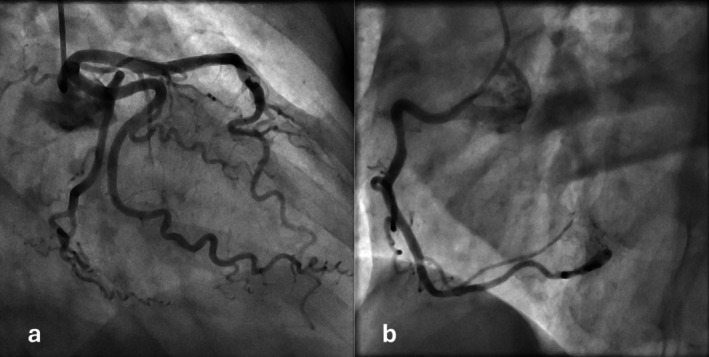
Coronary angiogram showing only mild coronary sclerosis (a: Left anterior descending and left circumflex artery; b: Right coronary artery).

The diagnosis of non‐ST elevation myocardial infarction (NSTEMI) was made and the patient was started on acetylsalicylic acid and was admitted to the intermediate care unit for heart rhythm monitoring. A coronary angiogram was performed, which showed only mild coronary sclerosis without any significant stenosis (Figure 3). Based on the collected diagnostic findings, a working diagnosis of TTS was made. Acetylsalicylic acid was discontinued, and Metoprolol 50 mg once daily was added to Valsartan as heart failure therapy. The patient's clinical condition improved fast, and she was subsequently discharged from the hospital after normalization of the QTc interval and was advised to have a follow‐up visit after 4 weeks with reassessment of myocardial function by TTE.

## 4 Conclusion and Results (Outcome and Follow‐Up)

**FIGURE 4:**
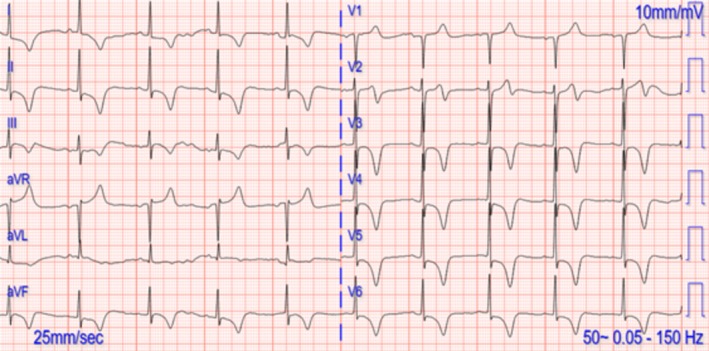
ECG on follow up visit with persistent marked T inversions.

**FIGURE 5:**
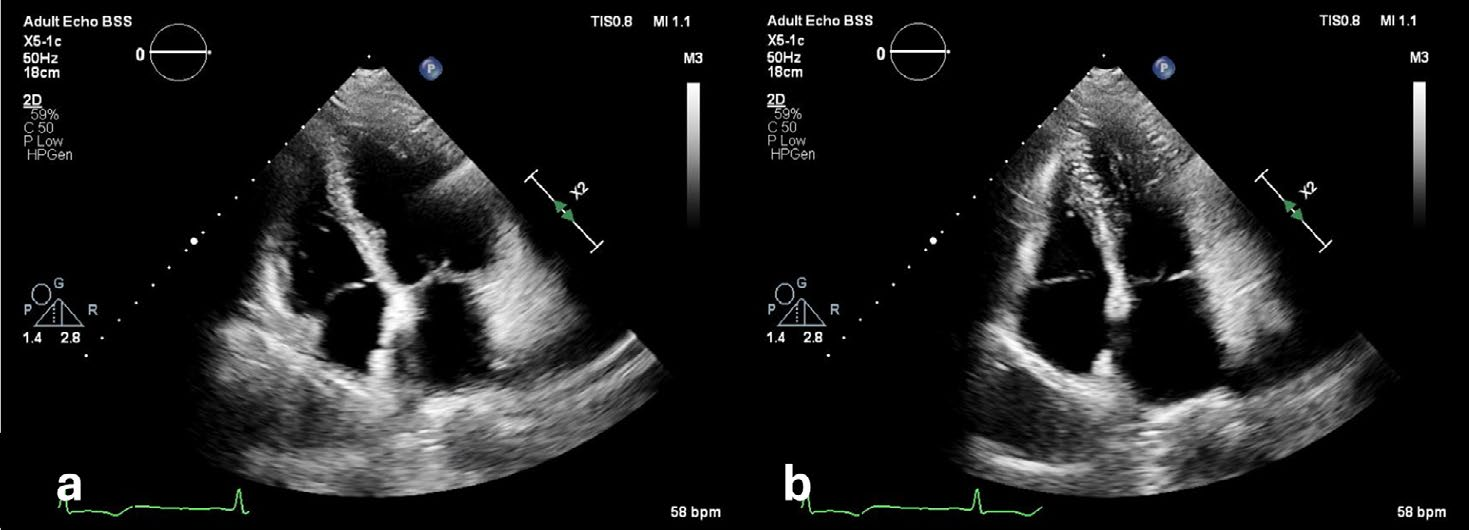
Apical four chamber view on follow up showing apical hypertrophy (a: End diastole; b: End systole).

Upon the follow‐up visit, the patient was asymptomatic and had normal vital signs. There was no significant clinical finding. Her ECG showed sinus rhythm with normal QTc interval, but persistent T wave inversions (Figure 4). The repeated TTE showed pronounced concentric apical hypertrophy consistent with an “Ace of Spades” appearance and a preserved LVEF of 70% and no residual regional wall motion abnormality (Figure 5). The normalization of LVEF supported the initial diagnosis of TTS, but the new finding with apical hypertrophy was suspicious for an ApHCM. For validation of an ApHCM, the patient was sent for a cardiac magnetic resonance imaging (CMR). The CMR findings, conducted 3 months after the event, showed a normally sized LV with normal function (LVEF 57%) and a dilated left atrium. No signs of apical hypertrophy could be detected. The right atrium and ventricle were of normal size and function (RVEF 55%). There were no significant valvular abnormalities, pericardial or pleural effusions, and the aortic root was of normal caliber. T2 mapping was performed and showed no evidence of persistent myocardial edema. Late enhancement images showed no myocardial scarring, but there were increased native T1‐Mapping values and an increased extracellular volume (ECV) of 52%.

## 5 Discussion

TTS, or “Broken heart syndrome,” is a clinical condition characterized by the dilatation of the LV chamber and the appearance of apical ballooning resembling a “Japanese fishing pot” as a result of sudden emotional or physical stress [1]. Although the precise mechanism remains unexplained, the most widely accepted explanation is a sudden surge of catecholamines that leads to myocardial injury [3]. The majority of those afflicted are women; in fact, 89.8% of those are female, according to research by Templin et al. [4]. The majority of patients are postmenopausal women with a mean age of 67 years, as in our case. A family history of ischemic heart disease is often present and is associated with favorable outcomes [5].

ApHCM is a variant of hypertrophic cardiomyopathy (HCM), and preferential thickening of the apical wall regions makes it stand out among other forms of HCM. Patients are usually diagnosed incidentally with pathologic routine ECG or when a TTE is performed for other heart conditions. The age at presentation is usually in the mid‐40s [6].

Among the many possible ECG findings in TTS are elevations in the ST segment in the anterior leads. Additionally, dynamic changes to ST‐T segments have been documented. The evolution to deep T wave inversions from elevated ST segments with marked QTc prolongation is noteworthy [7]. This evolution often occurs within 24 h of beginning and can persist for extended periods. In our case, the patient also presented with deep T wave inversions in anterior leads that persisted over a follow up of 4 weeks. ApHCM also presents with deep T wave inversions and therefore TTS‐related transient ECG changes can mimic or mask the ECG changes of ApHCM [8]. This similarity can make it difficult to establish a diagnosis, especially when the history and presentation are not considered appropriately.

TTE can help differentiate the two conditions at this point. In TTS, the LV shows apical ballooning, whereas the ApHCM appears as an “Ace of Spades” apical hypertrophy with possible obliteration of the LV apex [9, 10]. The ejection fraction in TTS is usually reduced with predominantly apical wall hypokinesia, while also midventricular or basal forms have been described [11]. ApHCM, on the other hand, shows a dynamic LV. In our case, the initial TTE showed ballooning of LV apex with a reduced LVEF of 35%; however, follow‐up TTE was consistent with the findings seen in ApHCM. This misinterpretation could be due to apical edema mimicking hypertrophy.

Coronary angiography is indicated in suspected TTS to rule out AMI. In these cases, the epicardial coronary arteries are usually free from any significant atherosclerosis or thrombi‐occlusions, which includes the condition to the group of myocardial infarction with nonobstructive coronary artery disease (MINOCA) [12, 13]. The left ventriculogram at this point usually shows the pot‐like LV with typical LV apical ballooning. ApHCM is also not considered a risk factor for CAD. According to current evidence, CAD is present in 6% of patients with ApHCM [14]. The “Ace of spades” LV can be seen during left ventriculography [15]. Similarly, in our case, no significant CAD was seen during the angiography. Apical ballooning was seen as consistent with TTS.

**FIGURE 6:**
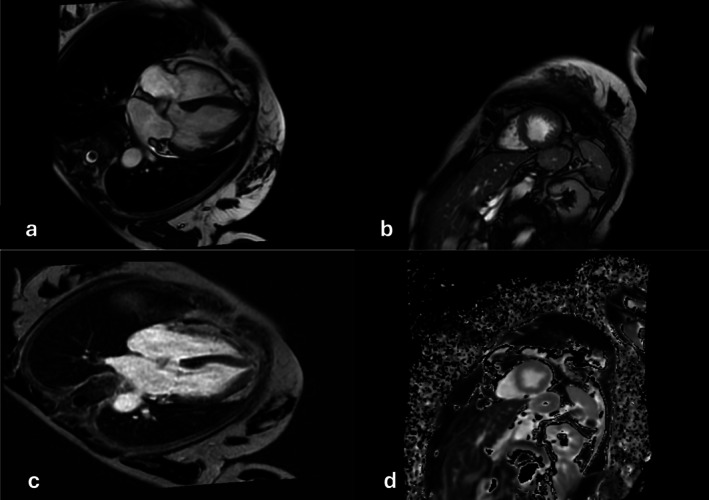
CMR showing normal apical LV wall thickness, no late gadolinium enhancement, and no edema (a: 4 chamber view; b: Apical short axis; c: Post contrast; d: T1 map).

CMR is the gold standard for diagnosing most structural heart diseases. Through tissue characterization it can help differentiate different types of cardiomyopathies. CMR helps to define the LVEF and the regional wall motion abnormalities. It can also be helpful to distinguish between reversible and irreversible myocardial injury. Complications of TTS, e.g., LV outflow tract obstruction and LV thrombus, can also be recognized by CMR and used for further management decisions [16]. CMR in ApHCM, on the other hand, shows apical LV thickening and possible obliteration of LV apex along with HCM typical late gadolinium enhancement of apex or LV apical aneurysm [17]. In our case, CMR showed no myocardial edema (Figure 6). However, T1‐Mapping values and ECV were elevated. While LGE imaging is the gold standard for detecting focal myocardial scarring, it is inherently limited in its ability to visualize diffuse interstitial fibrosis. This is particularly relevant in the context of TTS, where diffuse fibrosis has been reported to develop as early as 4 months in severe cases. Therefore, the use of ECV mapping in our study provides essential incremental value, as it may present diffuse fibrotic process that would otherwise remain undetected by conventional LGE sequences. At this stage, the future clinical course was uncertain; however, the patient remained completely free of symptoms.

Treatment recommendations for TTS are similar to those of heart failure. The condition is mostly reversible, especially in patients with an LVEF of > 35% at presentation. Most patients have improved or normalization of LVEF after 4–8 weeks. Nonetheless, 10% of these patients have adverse outcomes at long‐term follow‐up, as studied by Citro et al. [2]. ApHCM requires treatment similar to the other forms of HCM with beta‐blockers or calcium channel blockers in symptomatic patients. ICD may be indicated if risk factors are present. Rarely, surgery is needed [18].

Different diagnostic dilemmas make this case interesting for presentation. Firstly, the overlap between the ECG presentation of TTS and ApHCM makes the diagnosis challenging. Initial TTE findings suggestive of TTS were in contrast to the follow‐up echo findings. Gherbesi et al. reported a similar case where ApHCM initially remained undiagnosed due to TTS [19]. Recently, Maurizi et al. described a case where endocardial edema initially mimicked ApHCM and subsequently, the resolution of edema changed the diagnosis to TTS [20]. The two conditions can also coexist, as reported by Elhosseiny et al. in 2018 [21]. This highlights the dynamic presentation of the case, and the treating physicians need to be aware of the different presentations so that none of the conditions are missed.

Endocardial edema appears to be the leading player among all these different presentations. The edema can initially mask the apical thickness, and when it resolves over time, the underlying ApHCM can be appreciated more clearly. Vice versa, if edema is present in the LV apex it might give the impression of apical hypertrophy, as edema can persist up to 3 months after initial presentation [22, 23]. CMR plays a central role in this differentiation and is therefore recommended by current guidelines for the diagnostic workup of MINOCA [24]. However, it is very important that the examination is carried out promptly after diagnosis.

## Author Contributions

**Benjamin Vögeli:** conceptualization, visualization, writing – original draft. **Recep Ali Hacialioglu:** conceptualization, visualization, writing – original draft. **Bilal Cevik:** writing – review and editing. **Julian Flury:** writing – review and editing. **Alvin Oliver Payus:** writing – review and editing. **Nisha Arenja:** conceptualization, supervision, writing – review and editing.

## Funding

The authors have nothing to report.

## Consent

Written informed consent was obtained from the patient for publication of this case report and accompanying images.

## Data Availability

The authors have nothing to report.
